# Ten-Year Outcomes of Single-Anastomosis Sleeve Ileal (SASI) Bypass: Surgical Configuration Determines Long-Term Efficacy and Safety

**DOI:** 10.1007/s11695-025-08460-w

**Published:** 2026-01-28

**Authors:** Hosam Hamed, Mohamed Tarek, Waleed Gado, Mohamed Eldesoky, Tarek Mahdy

**Affiliations:** Department of Surgery, Mansoura University, Mansoura, Egypt

**Keywords:** Bariatric surgery, Obesity, morbid / surgery, Ileum / surgery, Weight loss, Diabetes mellitus, type 2 / surgery, Postoperative complications

## Abstract

**Introduction:**

The metabolic foundation of Single Anastomosis Sleeve Ileal (SASI) bypass is based on the combination of sleeve gastrectomy with a loop gastroileal bypass. The short- and mid-term outcomes were reported in favor of adequate weight loss and safe nutritional profile. The long-term efficacy and safety remain uncertain, particularly regarding optimal limb length and anastomosis size.

**Methods:**

A retrospective cohort study of patients who underwent SASI bypass between 2013 and 2014. Three groups based on the surgical technique: Group1: 2.5 m ileum, 4–6 cm anastomosis, Group2: 3.0 m ileum, 2.5 cm anastomosis, and Group3: 3.5 m ileum, 2.5 cm anastomosis. The primary outcome included weight loss, diabetic remission, and nutritional deficiencies requiring intervention.

**Results:**

Ninety-five patients were eligible with 10-year follow-up data available for 89 (93.7%). The mean percentage of excess weight loss (%EWL) was 58.6 ± 20.3%. Diabetic remission was sustained in 88%. Group1 had a 60% malnutrition rate and 40% reversal rate. Group2 and group3 showed significantly fewer nutritional complications, with no surgical revisions in Group 3. In this group, all diabetic patients remained in complete remission at 10 years. Weight outcomes between Groups 2 and 3 were comparable, though Group 3 had a slightly higher mean %EWL at 10 years (62.5% vs. 57.0%; p = 0.21).

**Conclusion:**

SASI bypass offers durable long-term weight loss and diabetic remission rates at 10 years. Outcomes are highly dependent on surgical technique. The current data suggests the optimal outcome is offered with the 3.5 ileum/ 2.5 cm anastomosis configuration. Standardization of technique is critical to ensure long-term efficacy and safety.

## Key Points

Surgical configuration dictates SASI’s long-term safety and efficacy.

Optimized SASI (350 cm/2.5 cm) yields durable weight loss and T2DM remission.

Short common channel (250 cm) caused severe malnutrition and high reversal rates.

Standardization of SASI anatomy is essential to minimize nutritional risk.

## Introduction

Obesity and related metabolic disorders remain a major global health concern. Among the current treatment options, bariatric surgery has demonstrated superior long-term outcomes in sustained weight loss and resolution of comorbidities [1].

Roux-en-Y gastric bypass (RYGB) was the most frequently performed bariatric procedure until sleeve gastrectomy (SG) came to the fore in 2013 [2]. However, both procedures have limitations. SG may be associated with suboptimal long-term weight maintenance and recurrence of type 2 diabetes mellitus, while RYGB involves greater technical complexity and an increased risk of nutrient malabsorption [3, 4].

The search for an optimal bariatric procedure, balancing efficiency, technical simplicity, and long-term safety, is still ongoing. In pursuit of a more effective yet simplified option, the Single Anastomosis Sleeve Ileal (SASI) bypass has emerged. It entails SG with a single-loop ileal bypass without duodenal exclusion, promoting both restrictive and metabolic effects [5, 6].

Early studies report promising results. A multicenter cohort of 551 patients showed 64% excess weight loss (%EWL) and 83.9% T2DM remission at one year [7]. In a recent meta-analysis comparing SASI bypass to SG, the former was associated with improved weight loss (MD = 11.32; 95% confidence interval (95%CI) [7.89;14.76]; *p* < 0.0001), and improvement or remission in T2DM (RR = 1.35; 95%CI [1.07;1.69]; *p* = 0.011), with similar complication rate [8].

However, concerns regarding long-term nutritional safety have emerged. Reports of protein-energy malnutrition, despite using a 300 cm common channel, have led to revisional surgeries in recent series [8–10]. While most available data cover only 1–3 years of follow-up, with few extending to four years, long-term outcomes remain poorly defined.

This study provides the first reported 10-year follow-up of patients undergoing SASI bypass. It also offers new insights into its durability, safety, and metabolic efficacy in relation to various surgical configurations of the procedure.

## Materials and Methods

### Study Design and Definitions

This is a retrospective cohort study of all patients who underwent Single Anastomosis Sleeve Ileal (SASI) bypass in the duration between January 2013 to December 2015 at a tertiary university-affiliated center. All patients underwent SASI Bypass as part of an IRB-approved prospective evaluation of one standardized metabolic procedure. Patients met NIH criteria for bariatric surgery and had no contraindications to standard procedures such as SG, RYGB, or OAGB. The universal application of SASI allowed uniform assessment of long-term outcomes and configuration-dependent refinements.

Eligible candidates for bariatric surgery were psychologically stable individuals with morbid obesity, aged between 18 and 65 years. All patients provided written informed consent after receiving a comprehensive explanation of the surgical procedure, including anticipated outcomes and the critical importance of long-term follow-up and lifelong nutritional supplementation. The study was approved by the Institutional Review Board of the Faculty of Medicine [R.25.06.3206]. The SASI bypass was selected for all cases to ensure procedural uniformity and evaluate the long-term safety of one standardized configuration. The study was conducted under controlled innovation guidelines, with Independent Data Safety Monitoring Committee (DSMC) oversight and full multidisciplinary preoperative evaluation. All patients provided informed consent after detailed counseling regarding procedure rationale, risks, and alternatives.

This study was conducted between 2013 and 2015, when bariatric surgery indications were limited to BMI ≥ 40 kg/m², or ≥ 35 kg/m² with comorbidities, according to the NIH 1991 and IFSO/ASMBS criteria applicable at that time. Patients with BMI < 35 kg/m² were not considered surgical candidates under those guidelines. Morbid obesity was defined as a body mass index (BMI) greater than 40 kg/m², or between 35 and 40 kg/m² in the presence of obesity-related comorbidities, including type 2 diabetes mellitus (T2DM), hypertension, dyslipidemia, and obstructive sleep apnea syndrome (OSAS) [10]. Weight loss outcome was evaluated using percentage of excess weight loss (%EWL). The diagnostic criteria for T2DM were based on the guidelines of the American Diabetes Association [11]. Postoperative complications and remission of obesity-related comorbidities were classified according to the standardized outcome reporting criteria for bariatric surgery [12, 13].

The primary outcomes included %EWL and T2DM remission after 10 years. Secondary outcomes were nutritional deficiencies over the follow-up period.

### Preoperative Preparation

All patients underwent comprehensive evaluation by a multidisciplinary bariatric team. During the 2013–2015 enrollment period, preoperative endoscopy was performed selectively for symptomatic or high-risk patients, in accordance with then-current IFSO and ASMBS recommendations. Universal endoscopy was not standard practice at that time. All patients were screened for Helicobacter pylori infection using urea breath or stool antigen testing. Positive cases received standard triple therapy before surgery, with confirmation of eradication prior to operation. Patients with mild or medically controlled GERD (Los Angeles grade A–B) were not excluded, in accordance with the bariatric standards of the study period. SASI was selected after detailed counseling and informed consent.

Thromboembolic prophylaxis was administered to individuals with a prior history of thromboembolic events in accordance with established guidelines [14]. Patients diagnosed with obstructive sleep apnea syndrome (OSAS) were managed with continuous positive airway pressure (CPAP) therapy under the supervision of a pulmonologist. Metabolic abnormalities were corrected prior to surgery to the extent possible, although complete normalization was not achieved in some cases despite extended preparation. Active smokers were excluded from surgery until documented cessation for at least 6 weeks. Patients with a remote smoking history (> 6 months abstinent) were not excluded. No active smokers were operated upon in this series.

### Surgical Technique

All procedures were carried out laparoscopically by a single, experienced surgical team. The SASI bypass was executed based on the technique initially described by Mahdy et al. (2016), with subsequent refinements implemented during the study period [6]. The operation commenced with a standard sleeve gastrectomy performed over a 36-Fr bougie, extending from approximately 5–6 cm proximal to the pylorus. A loop gastroileostomy was then created, connecting the gastric antrum to a distal ileal segment measured retrogradely from the ileocecal valve using a calibrated tape. The gastro-ileal anastomosis was constructed on the anterior wall of the antrum, 6–8 cm from the pylorus, in an isoperistaltic, antecolic orientation. The anastomosis was side-to-side, with a calibrated orifice of 2.5–3 cm in the final configuration. This positioning minimizes bile reflux. The total bowel length was measured routinely.

Three configurations of the common channel length were employed during the study period. The first configuration (Group 1) entailed an ileal loop that was anastomosed 250 cm proximal to the ileocecal valve, creating a wide gastroileal stoma approximately 4–6 cm in diameter. This approach reflected the initial experience and was modeled after the Santoro technique.

The initial SASI configuration (250 cm common channel, 4–6 cm anastomosis) was modified during the study under IRB approval as part of an ethically supervised refinement process consistent with the IDEAL framework. Subsequent cohorts (300–350 cm common channel, 2.5 cm anastomosis) were introduced after interim safety review demonstrated the need to reduce malabsorption. Each modification was prospectively documented and performed following renewed patient consent.

In the second configuration (Group 2), the anastomosis was performed 300 cm from the ileocecal valve, with a narrower stoma (2.5 cm), achieved by hand-suturing the stapled enterotomy to restrict its diameter. This adjustment was introduced to mitigate early nutritional deficiencies observed in Group (1) The third configuration (group 3) entailed an anastomosis that was placed 350 cm from the ileocecal valve, maintaining the same 2.5 cm stoma diameter as Group (2) This later configuration, adopted in late 2015, aimed to further reduce the risk of malabsorptive complications while preserving efficacy.

All anastomoses were completed with linear staplers followed by hand-sewn closure of the enterotomy using a two-layer technique with 3 − 0 absorbable monofilament (PDS) sutures. Non-absorbable materials were not used to reduce the risk of chronic irritation and marginal ulceration. Intraoperative methylene blue leak testing was routinely performed.

## Postoperative Follow-up and Data Collection

All patients received proton pump inhibitor prophylaxis (omeprazole 40 mg daily or equivalent) for six months postoperatively, extended to 12 months in patients with reflux symptoms or prior ulcer history. All patients were prescribed standard post-bariatric supplementation, including daily multivitamins, iron, calcium/vitamin D, and vitamin B₁₂ as needed. A protein intake of 60–80 g/day was advised. Follow-up was conducted at 3, 6, and 12 months, and annually thereafter. At each visit, body weight and BMI were recorded. Percentage excess weight loss (%EWL) was calculated. Significant weight regain was defined as ≥ 25% of the weight lost from the post-surgical nadir. All data were recorded in a prospectively maintained database.

Postoperative reflux symptoms were evaluated using the GERD-Q questionnaire and confirmed endoscopically when indicated. Bile reflux was assessed through symptom inquiry, upper endoscopy, and biopsy in selected cases. 24-hour pH-metry was performed in persistent cases to differentiate acid from bile reflux. Endoscopic surveillance was performed routinely at 1 and 2 years postoperatively in the initial cohort (Group 1). In later groups, endoscopy was conducted selectively for symptomatic or high-risk patients, in accordance with institutional and IFSO recommendations. Overall, 44% of patients underwent postoperative endoscopy within two years.

Postoperative reflux improvement or resolution was evaluated at 6, 12, and 24 months using the same clinical and endoscopic criteria. Marginal ulcers were diagnosed by upper endoscopy in symptomatic patients or during scheduled surveillance endoscopy at one year. Ulcers were defined as mucosal disruptions at the gastro-ileal anastomosis; all were managed medically. Postoperative surveillance for H. pylori was conducted using urea breath or stool antigen testing at 12–24 months or when symptoms indicated. Positive cases received standard triple or quadruple eradication therapy with confirmatory testing after treatment.

T2DM remission was assessed per ASMBS guidelines [15] where complete remission was considered when HbA1c < 6.0% and fasting glucose < 100 mg/dL off medications, partial remission on improved glycemic control with reduced therapy, and no remission with persistent need for equivalent or intensified therapy. Hypertension, dyslipidemia, and OSA were considered resolved if no longer required treatment and presenting with normal measurements (e.g., blood pressure < 130/85 mmHg without medication).

Nutritional surveillance involved annual laboratory testing for hemoglobin, ferritin, iron, vitamin B₁₂, folate, calcium, vitamin D, albumin, and total protein, as well as clinical evaluation for signs of deficiency. Nutritional complications requiring medical or surgical intervention (e.g., intravenous supplementation, surgical revision, or reversal) were tracked. Bariatric revisions conducted at external centers were reviewed if available.

### Statistical Analysis

Continuous variables are presented as mean ± standard deviation, while categorical data are reported as frequencies and percentages. Pre- and postoperative comparisons were conducted using paired t-tests. Differences among the three groups were analyzed using one-way analysis of variance (ANOVA), followed by Tukey’s post-hoc test for pairwise comparisons. Categorical outcomes were evaluated using the Chi-square test or Fisher’s exact test, as appropriate. A two-tailed p-value of < 0.05 was considered indicative of statistical significance. All statistical analyses were performed using SPSS software, version 26.0 (IBM Corp., Armonk, NY).

## Results

### Study Population and Baseline Characteristics

During the study duration, a total of 102 patients underwent SASI bypass. Seven patients were excluded: six due to loss of follow-up before the 10-year timepoint, and one due to unrelated mortality in the eighth postoperative year (myocardial infarction). Accordingly, 95 patients (70 women, 25 men) were included in the final analysis. The mean preoperative weight is 126.3 ± 21.5 kg and the mean BMI is 45.3 ± 6.8 kg/m^2^. The mean age at surgery was 41.8 ± 9.7 years. Baseline comorbidities included type 2 diabetes mellitus (T2DM) in 51 patients (52.6%), hypertension in 44 patients (46.3%), dyslipidemia in 36 patients (37.9%), and obstructive sleep apnea (OSA) in 18 patients (18.9%). Smoking history was recorded for all patients. Active smokers were required to cease smoking ≥ 6 weeks before surgery; only verified abstinent patients proceeded. Former smokers (abstinent ≥ 6 months) were distributed as follows: 13% in Group 1, 17% in Group 2, and 12% in Group 3. No active smokers underwent surgery (Table 1).

**Table 1:** Baseline characteristics and early postoperative complications [BMI: body mass index, T2DM: type II diabetes Mellitus]

Group Patients (*N*)	Baseline BMI (kg/m²)	BMI Range (Min–Max, kg/m²)	T2DM Prevalence (*n*= %)	Early Nausea/Vomiting (*n*= %)	Wound Infections (*n*= %)	Ulcers (Year 1) (*n*= %)	Former smoker
Group 1 (15)	47.0 ± 7.2	38.2–58.7	10 (60%)	8 (53%)	2 (13%)	1 (7%)	2 (13%)
Group 2 (30)	44.8 ± 6.0	37.6–59.4	16 (53%)	0	1 (3%)	1 (3%0	5 (17%)
Group 3 (50)	45.0 ± 6.9	35.9–57.2	25 (50%)	0	0	0	6 (12%)
P value	0.47		0.32	< 0.001	0.18	0.5	0.8

All patients successfully underwent laparoscopic SASI bypass with the allocated technique. There were no intraoperative complications in this series and no 30-day postoperative anastomotic leaks or major surgical complications. The average total small bowel length in this cohort was estimated intraoperatively at 550–700 cm.

## Surgical Technique and Early Postoperative Complications

According to the surgical technique, the patients were divided into three groups. Fifteen patients underwent the 250/4–6 cm configuration. Thirty patients underwent the 300/2.5 cm configuration, and fifty patients underwent the 350/2.5 configuration. The total small bowel (TSB) was measured intraoperatively in all patients, yielding a range of 550–700 cm (mean 620 ± 41 cm). Based on these measurements, the proportion of the bowel excluded from nutrient flow differed across configurations. In Group 1 (250 cm common channel), approximately 60% of the small bowel was bypassed; in Group 2 (300 cm common channel), the bypassed proportion was 52%; and in Group 3 (350 cm common channel), 44% of the bowel was bypassed.

Patient baseline characteristics and early postoperative complications are summarized in Table:1. Two patients (2.1%) had marginal ulcers within the first year (one in Group 1, one in Group 2), one was symptomatic (epigastric pain + anemia) and one asymptomatic, detected during routine surveillance endoscopy. All healed with medical therapy, and healing was confirmed endoscopically.

### One-Year Weight Loss and Metabolic Outcomes

At one year postoperatively, weight loss outcomes were satisfactory across all groups. The mean percentage of total weight loss (%TWL) was 33.7 ± 8.4%, corresponding to an overall percentage of excess weight loss (%EWL) of 84.5 ± 16.2%. Group 1 demonstrated the highest mean %EWL at 92.0 ± 14.5%, followed by Group 2 at 86.3 ± 15.0% and Group 3 at 80.1 ± 16.8%. Although Group 1 exhibited superior early weight loss, these differences did not reach statistical significance (*P* = 0.09).

Among the 51 patients with type 2 diabetes mellitus (T2DM), 45 (88.2%) achieved complete remission, while the remaining 6 exhibited significant glycemic improvement, requiring reduced pharmacologic therapy. The mean HbA1c level in diabetic patients decreased from 8.1% preoperatively to 5.5% at one year (*p* < 0.001). Hypertension resolved in 68% (30/44) of affected patients, dyslipidemia in 69% (25/36), and obstructive sleep apnea (OSA) in 72% (13/18).

Regarding gastroesophageal reflux disease (GERD), symptom resolution occurred in 70% (14/20) of patients with pre-existing reflux. However, two patients in Group 1 developed de novo GERD requiring medical management, likely related to bile reflux through the wider gastroileal anastomosis. No de novo cases of GERD were observed in Groups 2 or 3.

Nutritional laboratory markers at one year revealed modest changes without evidence of clinically significant malnutrition. Hemoglobin levels remained stable (13.5 g/dL preoperatively vs. 13.2 g/dL at one year; *p* = 0.10). A statistically significant decline in serum iron (88 ± 30 to 75 ± 28 µg/dL; *p* = 0.04) and ferritin (120 ± 60 to 80 ± 55 ng/mL; *p* = 0.01) was noted. Vitamin B₁₂ concentrations remained within normal limits for most patients, with five requiring parenteral supplementation. Folate and calcium levels showed no significant change, whereas vitamin D levels improved significantly, likely due to routine supplementation (25[OH]D increased from 22 ng/mL to 35 ng/mL; *p* < 0.001). A slight reduction in serum albumin was observed (4.3 ± 0.3 to 3.9 ± 0.4 g/dL; *p* = 0.005); however, no patient had hypoalbuminemia (< 3.5 g/dL) or clinical signs of protein-calorie malnutrition during the first postoperative year. There were no reported sleeve-related complications as kinks or stricture.

### Ten-Year Nutritional Outcome

Nutritional outcomes across the groups are detailed in Table 2. Group 1 patients experienced the highest burden of nutritional deficiencies. Within the first two postoperative years, signs of malabsorption were evident: 33% (5/15) developed chronic diarrhea and steatorrhea, 27% (4/15) presented with protein malnutrition (albumin < 3.0 g/dL), and 53% (8/15) had iron deficiency anemia refractory to oral supplementation, necessitating intravenous iron therapy. Despite intensive dietary counseling and supplementation, 6 patients (40%) ultimately required surgical reversal of the SASI bypass due to persistent malnutrition. These reversals were performed at a median of 18 months postoperatively (range: 12–36 months). Reversal surgery was performed laparoscopically by dismantling the gastro-ileal anastomosis with a linear stapler. One patient with refractory bile reflux underwent conversion to Roux-en-Y gastric bypass (150 cm alimentary, 60 cm biliopancreatic limb) without distal gastrectomy. All patients achieved full nutritional recovery.

**Table 2:** Ten-Year nutritional outcomes and reversal rates by SASI bypass configuration

Groups	Group 1	Group 2	Group 3	*P* value
Protein Malnutrition (N, %)	4 (27%)	0	0	< 0.01
Iron Deficiency Anemia (N, %)	8 (53%)	9 (30%)	3 (6%)	0.04
Vitamin B12 Deficiency (N, %)	5 (33%)	6 (20%)	2 (4%)	0.08
Reversal Rate Due to Nutrition (N, %)	6/15 (40%)	0	0	< 0.001

Following reversal, nutritional parameters improved significantly, including stabilization of albumin levels and weight. However, these patients subsequently experienced weight regain and recurrence of obesity-related comorbidities. Among the remaining nine Group 1 patients who retained the SASI anatomy, all exhibited at least one nutritional deficiency requiring long-term management, primarily iron or vitamin B₁₂ deficiency. Thus, every patient in Group 1 developed some form of deficiency, highlighting the high risk of malnutrition associated with the short common channel configuration in this group.

### Ten-Year Weight Loss Outcomes

**Fig. 1:**
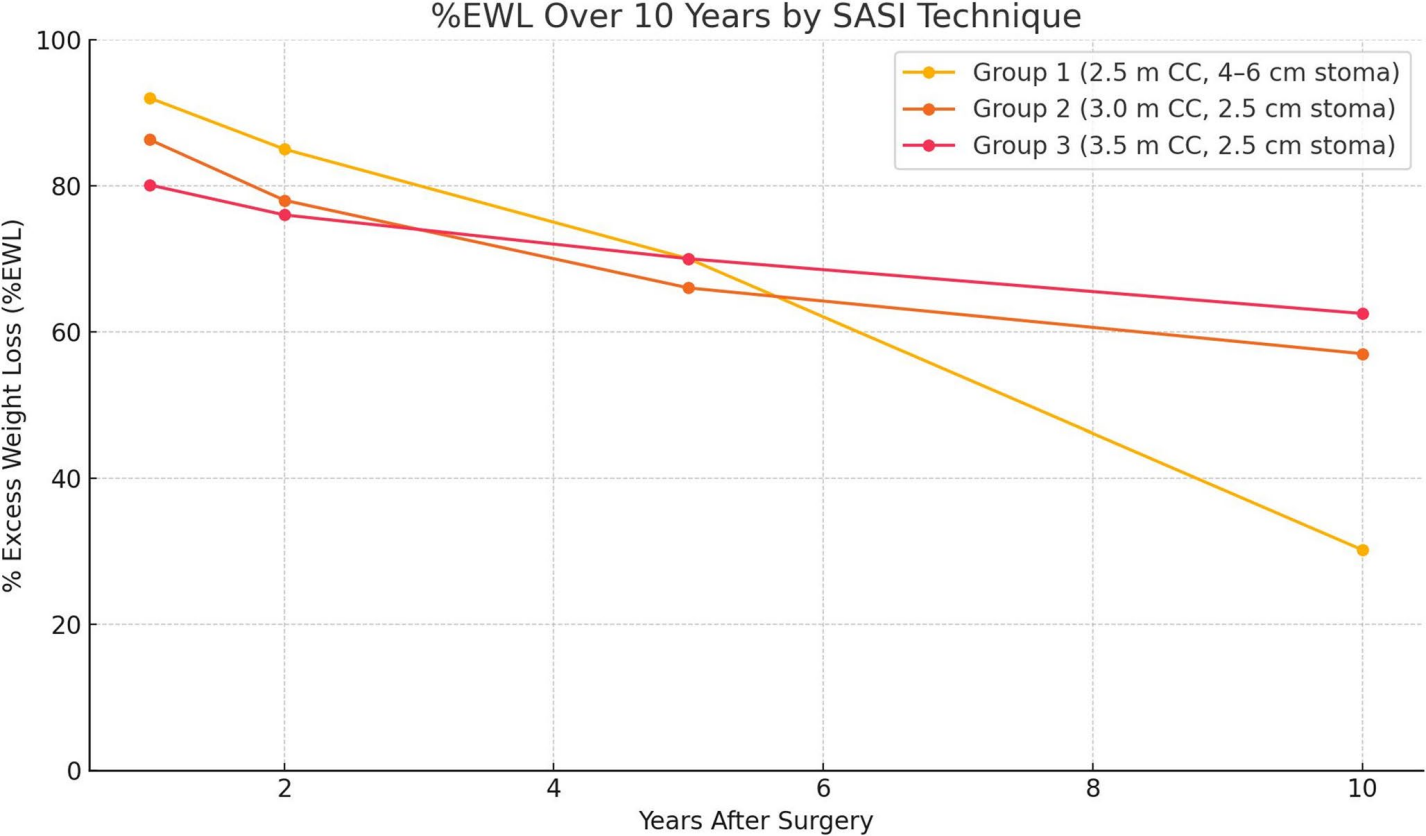
Ten-year weight loss outcomes following Single-anastomosis Sleeve Ileal (SASI) bypass. The graph depicts mean percentage of excess weight loss (%EWL) over time across the three surgical configurations: Group 1 (250 cm common channel, 4–6 cm anastomosis), Group 2 (300 cm, 2.5 cm anastomosis), and Group 3 (350 cm, 2.5 cm anastomosis). Group 1 showed higher early weight loss but was associated with severe malnutrition and a 40% reversal rate. Groups 2 and 3 maintained durable weight loss, with Group 3 achieving the most favorable long-term balance between efficacy and safety

Figure 1 illustrates the weight loss trajectory over 10 years, stratified by group. Patients experienced a sharp decline in weight during the first year, with stabilization or modest continued loss through year two, reaching a nadir BMI of 27.5 ± 4.3 kg/m² and mean %EWL of 90%. Thereafter, a gradual weight regain was observed. At 10 years, the mean BMI was 30.8 ± 5.7 kg/m² and mean %EWL was 58.6 ± 20.3%, reflecting an average regain of approximately 15% from nadir weight. Despite this, 74 of 89 patients (83.1%) maintained ≥ 50% EWL.

All six patients who underwent surgical reversal (Group 1) experienced significant weight recurrence, with mean %EWL falling to 12.5% and BMI rising to ≥ 35 kg/m² by year 10. Excluding these, the average %EWL among patients with intact SASI anatomy was 62.5%. Notably, none of the patients with a preserved bypass required conversion to another bariatric procedure, highlighting the long-term durability of SASI when the configuration is maintained. Table 3 summarized the %EWL, reversal rate, and number of patients with EWL > 50% among the three groups.

**Table 3:** Ten-year weight loss and metabolic outcome [%EWL: percentage of excess weight loss, T2DM: type II diabetes Mellitus]

Groups	Group 1	Group 2	Group 3	*P* value
10-Year %EWL	30.2 ± 25.7%	57.0 ± 18.9%	62.5 ± 17.5%	< 0.001
Reversal Rate (N, %)	6 (40%)	0	0	< 0.001
≥ 50% EWL at 10y (N, %)	9 (60%)	21 (70%)	44 (88%)	0.02
T2DM Patients (N, %)	10 (60%)	16 (53%)	25 (50%)	
T2DM Remission (N, %)	5 (50%)	14 (87.5%)	25 (100%)	0.001
Hypertension Remission (N, %)	6/12 (50%)	11/16 (69%)	14/18 (78%)	0.15

### Ten-Year Metabolic Outcomes

Out of 51 patients with preoperative T2DM, 45 (88.2%) were in complete remission at 10 years (no medication, normal glycemic indices). This overall rate mirrors the 1-year remission, indicating minimal late relapse overall. Regarding hypertension, 31/44 patient (70%) of hypertensive patients had normal blood pressure without medications at 10 years. Intergroup variations in diabetic remission and hypertension resolution are summarized in Table 3. For dyslipidemia, of 36 with dyslipidemia, 28 (78%) had remission (normal lipid profile without drugs) at 10 years [85% in Group 3, 75% in Group 2, and 50% in Group 1; the latter often resumed statins post-reversal]. Regarding OSA, 12 of 18 patients (67%) remained free of OSA symptoms at 10 years; others with weight regain had OSA return.

Preoperative GERD was present in 12 patients (12.6%). GERD resolved in 83%, improved in 17%, and new mild reflux developed in 4 patients (all Group 1). No GERD occurred in the later configurations (Groups 2 and 3). Bile reflux was assessed clinically and confirmed by endoscopy showing bile pooling and mucosal irritation. Three cases (3.1%) of bile reflux were documented—all in Group 1 (250 cm common channel, wide anastomosis). Bile reflux occurred in 3 patients (3.1%), all in Group 1 (short common channel, wide anastomosis). None occurred in Groups 2 or 3. Acid reflux improved in 83% of patients with preoperative GERD, while 4 developed new-onset mild reflux.

## Discussion

Early evidence suggests that SASI bypass provides durable long-term weight loss on par with established bypass procedures. Short- and mid-term studies have shown SASI achieving % excess weight loss comparable to or exceeding that of OAGB and RYGB [16–21]. These findings imply that SASI’s combined restrictive and malabsorptive mechanism can sustain weight reduction with a lower risk of late weight regain relative to purely restrictive procedures like sleeve gastrectomy [8]. Overall, SASI’s weight-loss durability appears to approximate that of RYGB and OAGB over the long term [21]. Compared to RYGB, the SASI bypass involves a single, tension-free gastro-ileal anastomosis and does not require jejunojejunostomy or extensive mesenteric division. For surgeons familiar with sleeve gastrectomy, this configuration may offer a shorter operative time and reduced risk of internal hernia formation, while maintaining a comparable metabolic effect.

Also, this study offers important insights into the long-term safety profile and durability of the SASI bypass, emphasizing the impact of surgical configuration on outcomes. A key strength lies in the direct comparison of configurations differing in common channel length and anastomosis size. Our findings demonstrate that a 350 cm common channel combined with a narrow 2.5 cm gastroileal anastomosis provides the most favorable balance, achieving substantial weight loss and durable metabolic outcomes while minimizing nutritional complications. In contrast, the initial configuration with a 250 cm limb and wide anastomosis was associated with a high incidence of protein-calorie malnutrition and a 40% reversal rate, highlighting the risks of excessive malabsorption. These results support the need to standardize SASI to a more moderate anatomical construct specifically, a 300–350 cm common channel with a small-caliber stoma to ensure long-term safety and reproducibility.

The SASI bypass functions through dual-pathway nutrient flow, allowing chyme to pass either through the pylorus or the gastro-ileal loop, where early ileal exposure enhances GLP-1 and PYY secretion. In Group 1, the wide 4–6 cm anastomosis caused preferential flow into the ileal limb, effectively bypassing the pylorus. Combined with a short 250 cm common channel, this produced a physiologic effect similar to a distal bypass and resulted in excessive malabsorption, including diarrhea, protein-energy malnutrition, and a 40% reversal rate. This differs from SADI-S, where the duodenum and proximal jejunum are preserved, maintaining a larger absorptive surface before the common channel. In SASI Group 1, direct diversion of nutrients from the antrum to the distal ileum created a greater functional bypass than the anatomic limb length alone would suggest. Refining the technique to a narrow 2.5 cm stoma and a 300–350 cm common channel restored physiologic balance and eliminated severe malnutrition.

Although Group 1 exhibited higher malnutrition rates, the mean %EWL was paradoxically lower at long-term follow-up. This discrepancy reflects early reversals for malnutrition, higher baseline BMI, and unbalanced malabsorption from a shorter common channel. Severe protein depletion likely limited sustainable fat loss, explaining the weaker long-term EWL. After standardizing supplementation and lengthening the common channel to 350 cm, both malnutrition and suboptimal weight loss were eliminated.

Long-term weight outcomes reinforce SASI’s efficacy, particularly in the optimized configuration. Patients in Group 3 (350 cm limb) achieved a mean %EWL of 62% at 10 years, with a corresponding BMI reduction from 45 kg/m^2^ to approximately 30 kg/m^2^. This performance is comparable to or exceeds that of RYGB, and aligns with published OAGB data reporting 68–75% EWL at 10 years. Notably, no patient with an intact SASI required conversion for weight failure, contrasting with the higher revisional rates reported after sleeve gastrectomy.

Weight regain occurred primarily between years 3 and 10, averaging 15% of initial weight, and was mostly limited in magnitude. The subgroup with significant regain (> 25% of lost weight) was predominantly composed of patients who had their bypass reversed, underscoring the importance of maintaining the anatomical alteration. Group 3’s modest early weight loss trajectory may have contributed to more stable long-term outcomes, consistent with the concept that gradual, sustained weight loss is associated with reduced physiological counter-regulation and lower relapse risk.

Early versions of the SASI procedure raised concern for malnutrition. Patients who underwent the initial SASI configuration (with a 250 cm common channel and a wide gastroileal anastomosis) experienced a high incidence of protein-calorie malabsorption and micronutrient deficiencies, akin to a very distal bypass. In our series, this overly short common channel led to chronic diarrhea, refractory iron-deficiency anemia, and hypoalbuminemia in a substantial subset, ultimately necessitating surgical reversal in 40% of those patients. This aligns with the alarming findings by Wafa et al., who reported severe malnutrition complications and a 20% revision (reversal) rate within just one year of SASI [10]. Fortunately, technical refinements have dramatically improved the nutritional safety of SASI. Adopting a longer common channel (approximately 300–350 cm) and a narrower gastroileal stoma (2.5 cm) preserves sufficient absorptive capacity while still providing significant weight loss and metabolic improvement.

The elimination of bile reflux in Groups 2 and 3 demonstrates the importance of limiting anastomotic diameter and extending the common channel—confirming that the refined SASI configuration can effectively prevent reflux-related complications. Although GERD is often considered a relative contraindication for sleeve-based operations, our data support that SASI—with optimized anastomotic calibration—can safely achieve reflux resolution in most patients, aligning with recent observations that the addition of a controlled gastro-ileal bypass mitigates high-pressure sleeve physiology.

Moreover, a recent meta-analysis noted that lengthening the common channel in SASI tends to reduce complications at only a minor cost to early weight loss, underscoring the importance of a balanced configuration [22]. Standardizing SASI to a moderately malabsorptive setup (approximately 3–3.5 m common channel with a small-caliber gastroileal anastomosis) can maximize long-term efficacy while minimizing nutritional risks. This standardized configuration is aimed at ensuring sustained weight loss with an acceptable nutritional profile [23, 24]. Notably, SASI can be readily reversed (by takedown of the gastro-ileal loop) if malnutrition or excessive weight loss occurs [8]. Such reversals, however, often lead to partial weight regain as the malabsorptive component is removed, underscoring the critical role of the bypass limb in maintaining long-term weight outcomes.

Iron-deficiency anemia occurred more frequently in Group 2 (20%) than in Group 3 (4%, *p* = 0.038). This difference likely reflects configuration-related malabsorption from a shorter common channel, inconsistent early supplementation adherence, and recurrent H. pylori infection in a minority of patients. After implementing structured nutritional follow-up and extending the common channel to 350 cm, iron deficiency was nearly eliminated, emphasizing the importance of standardization in limb length and postoperative supplementation protocols.

Compared with SADI-S and OAGB, the Single-Anastomosis Sleeve Ileal (SASI) Bypass in this series achieved equivalent long-term weight loss and superior nutritional safety, with lower rates of marginal ulceration and bile reflux. Unlike SADI-S, SASI preserves pyloric function and avoids complete duodenal bypass, thereby reducing fat-soluble vitamin and protein deficiencies while maintaining full metabolic efficacy [25, 26].

At 10 years post-SASI, approximately 88% of patients with preoperative type 2 diabetes remained in remission, demonstrating remarkably durable glycemic control. This long-term remission rate appears higher than that reported for Roux-en-Y gastric bypass (RYGB), where only about one-third to one-half of patients maintain diabetes remission by a decade. It is also comparable to the excellent durability seen with the biliopancreatic diversion/duodenal switch (BPD-DS), which can sustain T2DM remission in about 68–85% of cases at 10 years [27].

The sustained type 2 diabetes remission observed in SASI patients, particularly the 100% remission rate in Group 3 at 10 years, is likely multifactorial. Key contributors include significant and durable weight loss (mean BMI 30 kg/m^2^), as well as the hormonal effects induced by nutrient delivery to the distal ileum, namely enhanced secretion of GLP-1, PYY, and altered bile acid metabolism, all of which promote improved insulin sensitivity and pancreatic function. These mechanisms have been shown to drive glycemic improvement independent of weight loss in other bypass procedures. The absence of relapse in Group 3 may reflect both anatomical stability (no reversals) and consistent distal gut stimulation. Although one might assume that a longer common channel (3.5 m) reduces metabolic efficacy by limiting malabsorption, our data suggest otherwise; Group 3 achieved superior glycemic outcomes compared to Group 2 (100% vs. 88% remission), likely due to comparable weight loss and sufficient hormonal activation despite the modest anatomical difference. This supports the concept that aggressive malabsorption is not required for sustained diabetes remission echoing findings in duodenal switch literature where lengthening the common channel preserved metabolic benefit while improving nutritional safety. Collectively, these findings underscore the metabolic effectiveness of an optimized SASI configuration.

Long-term weight-loss and metabolic results of SASI bypass appear generally comparable to Roux-en-Y gastric bypass (RYGB), with the added benefit of fewer micronutrient issues. At 10 years post-RYGB, patients typically maintain about 50–60% excess weight loss (%EWL) and roughly 30–50% of patients have sustained type 2 diabetes remission [28]. In our 10-year SASI cohort, weight loss efficacy was similar to RYGB, and Group 3 (standard SASI configuration) achieved durable weight loss with 0% incidence of iron or B12 deficiencies [29]. Compared to one-anastomosis gastric bypass (OAGB), the SASI bypass shows equivalent efficacy while potentially avoiding certain drawbacks of the OAGB [16]. Sleeve gastrectomy tends to achieve 50–55% EWL at 5–6 years on average [30], but weight regain is common beyond five years, diminishing its durability [31]. A recent meta-analysis found no significant differences in weight loss or metabolic improvement between SASI and OAGB and greater %EWL and higher diabetes remission rates than SG (e.g. 11% EWL and 35% higher T2DM remission in the mid-term) [8].

Our findings underscore the importance of standardizing the SASI bypass technique to optimize outcomes and minimize complications. Early variations in surgical approach, such as shorter common channels (200–250 cm) or larger anastomoses, were associated with increased nutritional risk and inconsistent results across centers. Based on our 10-year data, a configuration with a common channel of ≥ 300 cm (ideally 350 cm) and a 2.5–3 cm anastomosis appears to provide the best balance of efficacy and safety. This aligns with prior recommendations and systematic reviews, including Attia et al., which emphasized the need for consistent outcome reporting by limb length to improve comparability [32]. Centers reporting favorable outcomes have typically adopted this more moderate approach, while excessive malabsorption may explain early discontinuation of SASI at some institutions. Long-term success also depends on structured follow-up: even patients with optimal anatomy (Group 3) showed early declines in ferritin, highlighting the need for ongoing nutritional surveillance. We recommend routine lifelong supplementation with multivitamins, iron, and vitamin B12, similar to protocols for RYGB.

Although the refined SASI configuration demonstrated excellent long-term nutritional stability and absence of bile reflux, early experiences (Group 1) confirmed that inadequate calibration of the common channel and anastomotic diameter could result in malnutrition, bile reflux, and marginal ulcers. These complications were configuration-related and not intrinsic to the SASI concept itself. Once the limb length was increased to ≥ 300 cm and the anastomosis narrowed to 2.5 cm, all such adverse events were eliminated, and no new cases occurred over 10 years of follow-up apart from one case of marginal ulcer that was treated conservatively. Accordingly, our data should not be interpreted as evidence that SASI is universally risk-free, but rather that its long-term safety is attainable with strict adherence to standardized limb lengths and anastomotic calibration.

The present findings support the continued integration of SASI bypass into the bariatric/metabolic surgery repertoire. Its favorable long-term outcomes make it an appealing choice for patients with T2DM requiring durable metabolic control. Future studies should aim to validate these results in larger, multicenter cohorts, ideally through randomized trials comparing SASI to RYGB and OAGB with extended follow-up. Mechanistic investigations evaluating hormonal responses, insulin sensitivity, and nutrient absorption across different SASI configurations may clarify the relative contributions of malabsorption versus enteroendocrine modulation to its efficacy.

Our study is limited by its single-center, non-randomized design and a relatively small initial SASI group, which may affect generalizability. It is worth noting that a large series of the related single-anastomosis sleeve jejunal bypass (SAS-J) reported 87% EWL at 1 year and durable 5-year outcomes with no significant deficiencies, suggesting nutritional safety comparable to SASI [33, 34]. However, it remains unclear if SAS-J offers any outcome advantage over the SASI bypass. Finally, regardless of procedure, lifelong vitamin and mineral supplementation and rigorous long-term follow-up are recommended for all patients to ensure sustained success and detect any late nutritional issues.

## Conclusion

At 10 years postoperatively, the Single Anastomosis Sleeve Ileal (SASI) bypass demonstrates sustained weight loss, a high rate of durable type 2 diabetes remission, and minimal nutritional complications when performed with an optimized configuration (350 cm common channel and 2.5 cm gastroileal anastomosis). In contrast, an earlier, more malabsorptive SASI configuration (common channel 250 cm, wide anastomosis) resulted in severe malnutrition requiring reversal, underscoring the need for surgical standardization. These findings indicate that an optimized SASI can achieve metabolic outcomes comparable to those of gastric bypass while preserving the nutritional safety profile of sleeve gastrectomy. Our 10-year results support SASI as a safe, effective bariatric option, particularly in patients with metabolic syndrome. Further large comparative studies are warranted to validate these outcomes and establish the long-term role of SASI in bariatric surgery.

## Data Availability

The datasets generated and/or analyzed during the current study are not publicly available due to institutional policies and patient confidentiality. De-identified data may be made available from the corresponding author (Dr. Hosam Hamed, hosam-eldin@hotmail.com) upon reasonable request and subject to approval by the Institutional Review Board of Mansoura University.

## References

[1] Schauer PR, Bhatt DL, Kirwan JP, Wolski K, Aminian A, Brethauer SA, et al. Bariatric surgery versus intensive medical therapy for diabetes − 5-year outcomes. N Engl J Med. 2017;376(7):641–51. 10.1056/NEJMoa1600869. 28199805 10.1056/NEJMoa1600869 PMC5451258

[2] Angrisani L, Santonicola A, Iovino P, Formisano G, Buchwald H, Scopinaro N. Bariatric surgery worldwide 2013. Obes Surg. 2015;25(10):1822–32. 10.1007/s11695-015-1657-z. 25835983 10.1007/s11695-015-1657-z

[3] Lind R, Hage K, Ghanem M, Shah M, Vierkant RA, Jawad M, et al. Long-term outcomes of sleeve gastrectomy: weight recurrence and surgical non-responders. Obes Surg. 2023;33(10):3028–34. 10.1007/s11695-023-06730-z. 37464052 10.1007/s11695-023-06730-z

[4] Evenepoel C, Vandermeulen G, Luypaerts A, Vermeulen D, Lannoo M, Van der Schueren B, et al. The impact of bariatric surgery on macronutrient malabsorption depends on the type of procedure. Front Nutr. 2023;9:1028881. 10.3389/fnut.2022.1028881. 36712518 10.3389/fnut.2022.1028881 PMC9877414

[5] Santoro S, et al. Sleeve gastrectomy with transit bipartition. Surg Obes Relat Dis. 2012;8(6):835–43.

[6] Mahdy T. Single anastomosis sleeve ileal bypass: a novel metabolic surgery procedure. J Laparoendosc Adv Surg Tech A. 2016;26(12):1011–8.

[7] Mahdy T, et al. SASI bypass: multicenter experience with 551 patients. Obes Surg. 2020;30(1):105–13.

[8] Oliveira CR, Santos-Sousa H, Costa MP, Amorim-Cruz F, Bouça-Machado R, Nogueiro J, Resende F, Costa-Pinho A, Preto J, Lima-da-Costa E, Carneiro S, Sousa-Pinto B. Efficiency and safety of single anastomosis sleeve ileal (SASI) bypass in the treatment of obesity and associated comorbidities: a systematic review and meta-analysis. Langenbecks Arch Surg. 2024;409(1):221. 10.1007/s00423-024-03413-w. PMID: 39023536; PMCID: PMC11258063. 39023536 10.1007/s00423-024-03413-w PMC11258063

[9] Khalaf H, Hamed H. SASI bypass: hopes and concerns after two years. Obes Surg. 2022;32(8):2451–9. 10.1007/s11695-020-04945-y 32844276

[10] Wafa A, Bashir A, Cohen RV, Haddad A. The alarming rate of malnutrition after single anastomosis sleeve ileal bypass. A single centre experience. Obes Surg. 2024;34(5):1742–7. 10.1007/s11695-024-07192-7. 38532145 10.1007/s11695-024-07192-7

[11] American Society for Metabolic and Bariatric Surgery. Updated position statement on weight loss surgery for the treatment of type 2 diabetes mellitus. Surg Obes Relat Dis. 2010;6(5):573–85.

[12] American Diabetes Association. Diagnosis and classification of diabetes mellitus [published correction appears in Diabetes Care. 2010;33(4):e57]. Diabetes Care. 2010;33 Suppl 1(Suppl 1):S62-S69. 10.2337/dc10-S062 PMC2797383 20042775

[13] Brethauer SA, Kim J, El Chaar M, et al. Standardized outcomes reporting in metabolic and bariatric surgery. Obes Surg. 2015;25(4):587–606. 25802064 10.1007/s11695-015-1645-3

[14] Mechanick JI, Youdim A, Jones DB, et al. Clinical practice guidelines for the perioperative nutritional, metabolic, and nonsurgical support of the bariatric surgery patient–2013 update: cosponsored by American association of clinical Endocrinologists, the obesity society, and American society for metabolic & bariatric surgery. Obes (Silver Spring). 2013;21(0 1):S1–27. 10.1002/oby.20461 PMC4142593 23529939

[15] American Society for Metabolic and Bariatric Surgery (ASMBS). Updated position statement on weight loss surgery for the treatment of type 2 diabetes mellitus. 2009. Available from: https://asmbs.org/resources

[16] Kermansaravi M, Kabir A, Pazouki A et al. 1-Year Follow-up of single anastomosis sleeve ileal (SASI) bypass in morbid obese patients: efficacy and concerns. Obes Surg. 2020. PMID 32681291. 10.1007/s11695-020-04781-0 32681291

[17] Emile SH, Mahdy T. Excessive Weight Loss and Hypoalbuminemia After SASI Bypass: the Need for Standardization of the Technique. Obes. Surg. 2021. PMID 32734570. 10.1007/s11695-020-04890-w 32734570

[18] Rezaei MT, et al. Single-anastomosis Sleeve Jejunal: a Mid-term Follow-up… [Journal] 2023. PMID 36847922. 10.1007/s11695-023-06520-7 36847922

[19] Hosseini SV, et al. Optimal Length of Biliopancreatic Limb in Single … Obes Surg. 2022. PMID 35583584. 10.1007/s11695-022-06107-8 35583584

[20] Liagre A, Benois M, Queralto M, Boudrie H, Van Haverbeke O, Juglard G, et al. Ten-year outcome of one-anastomosis gastric bypass with a biliopancreatic limb of 150 cm versus Roux-en-Y gastric bypass: a single-institution series of 940 patients. Surg Obes Relat Dis. 2022;18(10):1228–38. 10.1016/j.soard.2022.05.021. 35760675 10.1016/j.soard.2022.05.021

[21] Aghajani E, Schou C, Gislason H, Nergaard BJ. Mid-term outcomes after single anastomosis sleeve ileal (SASI) bypass in treatment of morbid obesity. Surg Endosc. 2023;37(8):6220–7. 10.1007/s00464-023-10112-y. Epub 2023 May 12. PMID: 37171643; PMCID: PMC10338567. 37171643 10.1007/s00464-023-10112-y PMC10338567

[22] Emile SH, et al. Systematic review of the outcome of single-anastomosis sleeve ileal bypass in treating morbid obesity with proportion meta-analysis of improvement in diabetes. Int J Surg. 2021;92:106024. 10.1016/j.ijsu.2021.106024. 34252597 10.1016/j.ijsu.2021.106024

[23] Hamed H, Ali M, Elmahdy Y. Types, safety, and efficacy of limb distalization for inadequate weight loss after Roux-en-Y gastric bypass: a systematic review and meta-analysis with a call for standardized terminology. Ann Surg. 2021;274(2):271–80. 10.1097/SLA.0000000000004485. 32941271 10.1097/SLA.0000000000004485

[24] Ugale S, Vennapusa A, Katakwar A, Ugale A. Laparoscopic bariatric surgery–current trends and controversies. Ann Laparosc Endosc Surg. 2017;2:92.

[25] Sánchez-Pernaute A, et al. Single-Anastomosis Duodeno-Ileal bypass with sleeve: Ten-Year outcomes. Obes Surg. 2021;31:2612–23.

[26] Carbajo MA, et al. Long-Term results of One-Anastomosis gastric bypass (OAGB): Weight, Metabolic, and nutritional outcomes. Obes Surg. 2019;29:456–64.

[27] Kapeluto JE, et al. Ten-year remission rates in insulin-treated type 2 diabetes after biliopancreatic diversion with duodenal switch. Surg Obes Relat Dis. 2020;16(11):1701–12. 10.1016/j.soard.2020.06.052. 32800734 10.1016/j.soard.2020.06.052

[28] Chahal-Kummen M, Eribe I, Stubhaug A, et al. Long-term results 10 years after laparoscopic Roux-en-Y gastric bypass: a prospective study. Surg Endosc. 2020;34(5):1923–33. 10.1007/s00464-019-07011-y.31312962

[29] Parkitna J, Binda A, Jaworski P, et al. Anemia and iron metabolism disorders after single-anastomosis sleeve ileal (SASI) bypass: is it a real problem? Langenbecks Arch Surg. 2024;409(1):195–204. 10.1007/s00423-024-03403-y. 38904793 10.1007/s00423-024-03384-y PMC11192672

[30] Shabbir A, Dargan D. The success of sleeve gastrectomy in the management of metabolic syndrome and obesity. J Biomed Res. 2015;29(2):93–7. 10.7555/JBR.28.20140107. 25859262 10.7555/JBR.28.20140107 PMC4389120

[31] Sabah SA, Haddad EA, Qadhi I, et al. Beyond the decade: unveiling long-term weight and co-morbidity outcomes up to 10 years post laparoscopic sleeve gastrectomy. Langenbecks Arch Surg. 2025;410(1):112. 10.1007/s00423-025-03680-1. 40163236 10.1007/s00423-025-03680-1 PMC11958372

[32] Attia S, et al. Single anastomosis procedures in metabolic surgery: a systematic review. Surg Obes Relat Dis. 2021;17(6):1150–60.

[33] Sewefy AM, Atyia AM, Mohammed MM, Kayed TH, Hamza HM. Single-anastomosis sleeve jejunal (SAS-J) bypass as a treatment for morbid obesity: technique and 6-year follow-up of 1986 cases. Int J Surg. 2022;102:106662. 10.1016/j.ijsu.2022.106662. 35568310 10.1016/j.ijsu.2022.106662

[34] Sayadishahraki M, Rezaei MT, Mahmoudieh M, Keleydari B, Shahabi S, Allami M. Single-Anastomosis sleeve jejunal Bypass, a novel bariatric Surgery, versus other familiar methods: results of a 6-Month Follow-up – a comparative study. Obes Surg. 2020. PMID 31768867. 10.1007/s11695-019-04266-9 31768867

